# Describing Non-Binary People’s Encounters with the Healthcare System: A Qualitative Study in Catalonia (Spain)

**DOI:** 10.1007/s10508-024-02849-x

**Published:** 2024-04-02

**Authors:** Rebeca Gómez-Ibáñez, Juan M. Leyva-Moral, Alexander Cruzado-Reyes, Lucas R. Platero, Nina Granel, Carolina E. Watson

**Affiliations:** 1 Nursing Department, Faculty of Medicine, Universitat Autònoma de Barcelona, Avinguda de Can Domènech, 08193 Bellaterra, Barcelona Spain; 2 Grupo de Investigación Enfermera Sobre Vulnerabilidad y Salud, Nursing Department, Universitat Autónoma de Barcelona, Bellaterra, Barcelona Spain; 3 Hospital Germans Tries I Pujol, Carretera de Canyet, Barcelona, Spain; 4 Department of Psychology, Rey Juan Carlos University, Madrid, Spain

**Keywords:** Non-binary people, Healthcare environment, Discrimination, Gender identity, Sexual orientation

## Abstract

Non-binary people face numerous stressors in their daily lives, including personal, interpersonal, and environmental. These stressors gain strength when such individuals access healthcare services, and discrimination and cisgenderism become the main barrier to obtaining gender-affirming healthcare. This study aimed to describe the experiences of non-binary people regarding the care and medical attention received in Catalonia (Spain). A qualitative phenomenological study was conducted with 21 non-binary people recruited using snowball sampling in 2022. Data were gathered through open-ended interviews and analyzed using thematic analysis. Two main themes were identified, which were further classified into two categories each: Theme 1—This is me composed of the categories, “My Name and My Pronouns” and “One’s Chosen Gender,” and Theme 2—I do not exist for the health system consisting of “Uneducated Health System in Sexual Health” and “Feeling Like an Outsider for Being Non-Binary.” Non-binary people face multiple stressors when accessing the healthcare services that makes them feel invisible, vulnerable, and marginalized. Further widespread implementation of person-centered care is essential to promote the relationship between non-binary people and the healthcare system. In addition, further sexual health training is required for all health professionals.

## Introduction

Gay, lesbian, bisexual, transgender, intersex, gender-diverse, and queer people have a presence in contemporary society that cannot be denied. Although evidence is limited in Spain, this diverse population faces various health issues stemming from social stigma and the denial of their rights (Ciria-Barreiro et al., 2021; Fernández-Garrido, 2017; García et al., 2020). In this study, we refer to non-binary people as those whose identity falls between or outside male and female identities, those able to experience both male and female identities at separate times, or someone who does not experience gender identity at all (Matsuno & Budge, 2017).

Estimating the prevalence of transgender and gender-diverse (TGD) people is challenging owing to the lack of data (Reisner et al., 2015). Estimates of the numbers of non-binary people vary substantially within the available literature, with a higher proportion of young people identifying as non-binary (Ballering et al., 2023; Jones et al., 2019; Kearns et al., 2021; Monro, 2019; Parra & Missé, 2022). Quantifying TGD people in research becomes difficult because data on gender identity is not routinely collected, neither in medical record systems nor population health surveys nor otherwise (Gil-Borrelli et al., 2017; Valentine & Shipherd, 2018). Additionally, this could be because transgender and gender non-conforming people often lack confidence in various government institutions due to the possibility of discrimination and may choose to hide their identity (Hansen & Żółtak, 2022; Kantor et al., 2023; Kcomt et al., 2020).

In Spain, studies suggest that the National Health Information System still contains scarce information regarding gender identity, leading to the invisibility of trans people in health statistics, not properly classifying people, distorting results, and even hindering the design of interventions (Parra & Missé, 2022). It is noteworthy that asking for personal data, such as one’s sexuality or gender identity, is not only a sensitive matter but also has a cultural specificity, given that Spain is a country in which asking for data, including ethical background or sexuality, is not commonly performed by public administrations. In turn, this lack of data entails discrimination as trans people are deprived of being registered by public administrations with their sense of gender-gender identity (Gil-Borrelli et al., 2017). Thus, this reality suggests that the health needs of vulnerable groups, outside of specialized research, are unaddressed (Fernández-Garrido, 2017).

Although there are individuals who have broken the norms assigned to their gender (Matsuno & Budge, 2017; Monro, 2019), people of non-binary gender are a minority that has not received adequate attention within the LGBTIQ+ collective (Hansen & Żółtak, 2022). Non-binary people face numerous stressors in their daily lives, such as personal (the result of the lack of resources and information to act based on their own gender identity and lack of family support), interpersonal (discrimination and exclusion of the LGBTIQ+ collective itself), and environmental (lack of representatives and adapted public spaces, for example, public toilets) (Fiani & Han, 2019; López-Gómez & Platero, 2018; Parra & Missé, 2022). In terms of accessing the healthcare system, these stressors gain strength, and discrimination and cisgenderism become the primary barriers in accessing gender-affirming healthcare (Wright et al., 2021). Institutional policies constrain their access to healthcare and may act as a kind of structural stigma, which strengthens the idea of gender binarism (McCann et al., 2021). A recent qualitative scoping review addressing TGD people’s experiences receiving healthcare attention not only found that most of their experiences and needs were related to challenges, including financial and insurance barriers, but also notably emphasized the lack of the providers’ knowledge and sensibility (understood as lack of competency, stigma, hostile treatment settings, among others) (Teti et al., 2021; Wickham et al., 2023). In some cases, healthcare was denied to them due to their gender identity (Kearns et al., 2021). All these barriers lead to adverse healthcare experiences for TGD people without responding to their medical needs.

Although stigma, discrimination, and marginalization experiences in accessing healthcare are felt and recognized by this population, positive experiences (Kearns et al., 2021) and key facilitators (McCann et al., 2021) have also been identified, mainly affirming attitudes from healthcare providers. TGD gender-affirming experiences hold the potential to regain autonomy and greatly impact attrition rates, thus improving the healthcare outcomes (McCann et al., 2021). Therefore, gender identity affirmation has shown to be an additional resilience factor for trans and non-binary people’s well-being (Doyle et al., 2021).

In the past five years, research has documented differences in health outcomes and rates of gender-affirming interventions among TGD individuals, although the findings remain inconclusive (Ballering et al., 2023; Cheung et al., 2020; Jones et al., 2019; Wickham et al., 2023). Further research is needed to better understand the experiences and needs of specific groups of TGD when accessing health services (McCann et al., 2021; Teti et al., 2021), specially focusing on the views, needs, and experiences of non-binary people (Cheung et al., 2020).

Scientific evidence to describe or explain non-binary daily life experiences in Spain is very limited ( Jacques-Aviño et al., 2021; López-Gómez & Platero, 2018; López-Gómez & Tobalina, 2022). When non-binary populations are included in research, they are most often merged with binary transgender populations. However, research shows that non-binary populations face even more discrimination and express worse physical and psychological health outcomes than binary trans people (Aparicio-García et al., 2018; Ciria-Barreiro et al., 2021; López-Sáez & Platero, 2022); as a result, we need a more nuanced and complex understanding of the trans category (Parra & Missé, 2022). Some non-governmental organizations have conducted specific interventions for trans and non-binary people, but they have not employed a scientific approach. Thus, in the Spanish context practice may be informed by evidence coming from studies conducted in different sociocultural contexts. Understanding current experiences is crucial to add evidence to the extant knowledge and improve the evidence base of their lived experiences, needs, and interventions required. To fill in this gap, this study aims to describe the experiences of non-binary people regarding the healthcare and medical attention received in Catalonia, since health care is a conflict zone for non-binary people in Spain (López-Gómez & Tobalina, 2022). The research question is how do non-binary people experience the healthcare and medical attention received in Catalonia?

## Method

This study employed a qualitative phenomenological study design. This type of research is beneficial when trying to interpret the essence of life experiences and recognize personal meanings with respect to the main topic of research (Fuster-Guillen, 2019).

### Participants

Snowball sampling was used to recruit participants to identify and select informants who could provide valuable information to the research (Palinkas et al., 2015). The principal investigator and other research members directly invited their personal contacts by email and WhatsApp. Moreover, personal social media accounts were used to promote the study. Finally, LGBTIQ+ specialized NGOs promoted the study among their clients. The inclusion criteria were as follows: self-identify as non-binary, be over 18 years old, and have experienced at least one care process in the health system in Catalonia during the last year. In contrast, the exclusion criteria were the following: presenting a severe language barrier or cognitive difficulties or mental health problems that hinders participation.

A total of 21 people were included in the study, with a mean age of 29.05 years and an age range of 19–43 years. The experiences of participants with the Catalonian health services have focused on primary care, with exception of two participants that discussed emergency care services. Table 1 summarizes the main sociodemographic characteristics of the participants. Table 2 shows the summary of themes, categories, and codes. Verbatim narrations are specified by including a pseudonym and the age of the person interviewed.

**Table 1:** Sociodemographic characteristics of the participants

	*N* (%)
*Nationality*	
Spain	14 (66.67)
Latin America	5 (23.81)
Northern Europe	1 (4.76)
Sub-Saharan Africa	1 (4.76)
*Level of studies completed*	
Basic	5 (23.81)
Secondary	9 (42.86)
University	7 (33.33)
*Occupation*	
Employed	15 (71.43)
Student	6 (28.57)
*Relationship status*	
Single	17 (80.95)
Married	3 (14.29)
Co-habiting	1 (4.76)
*Healthcare assistance reasons*	
Emergency	2 (9,5%)
Scheduled	19 (90,5%)

**Table 2:** Themes, categories, and codes

Themes	Categories	Codes
This is me	My name and my pronouns	Legal identification
		Pronoun
		Health documentation
	One’s chosen sex	Discomfort
		Classical dichotomy
		Obligation to define oneself
		Exclusionary anatomy
		Discomfort
		Incomprehension
		Choosing not to go to health centers
		Neutral public spaces
		Toilets
		Hospitalization
I don’t exist for the health system	Lack of education in sexual health in the health system	Misconceptions
		Feeling of having to teach
		Educational context
	Feeling like an outsider for being non-binary	Small Group
		Discrimination
		Give your place
		Normalizing diversity

### Measures and Procedure

Data were collected in 2022 using open-ended interviews (Jamshed, 2014) whose prompt question was as follows: “Tell me about your experiences with the public health care system.” To form the sample, activists linked to the LGBTIQ+ community were contacted to explain the study, its objective, and the possibility of inviting non-binary people as participants. Through these activists, the potential study participants were accessed. They were contacted by telephone and subsequently provided with detailed information about the project, thereby allowing them to resolve any doubts related to their participation. Finally, informed consent was obtained to proceed with the face-to-face anonymized interviews.

The interviews were conducted in private and comfortable spaces chosen by the participants. In phenomenology, a typical sample size should be around 12–15 people (Guest et al., 2006; Polit & Beck, 2017). However, in our study, we determined the number of participants based on theme saturation (Sandelowski, 2008), which occurred after interviewing 21 participants. The interviews were audio-recorded, with an average duration of 70 min. To ensure consistency and minimize potential bias in data collection, all interviews were conducted and transcribed by the principal investigator, aiming to maintain uniformity in the interview process and transcription quality. Any data that could reveal the identity of the participants was removed from the transcripts and pseudonyms were used instead of their real names. The main observations were summarized, such as the recurring themes mentioned during the interviews, sensations, difficulties, facilities, and reflections (Polit & Beck, 2017). Once the first analytical report was obtained, it was confirmed with the participants who did not propose any changes. The interviews were conducted in Spanish and translated into English by a professional bilingual translator. Participants did not receive any financial rewards for their participation.

### Data Analysis

The interview transcripts underwent thematic analysis according to Boyatzis’ (1998) approach. This coding process included several debriefing meetings to achieve consensus. Next, codes were grouped into four preliminary themes based on similarities and discrepancies. After carefully reflecting on and comparing the data, we classified the preliminary themes into two final themes. Finally, the phenomenon under study was described using the identified themes. Lastly, findings were verified conducting a member-check meeting with some participants, two qualitative methods experts and a psychologist specialized in gender identity issues (Morse et al., 2002).

## Results

### This Is Me

This theme refers to those aspects that participants emphasize as possible ways to forge their real identity, both to identify themselves and to be identified by others. This topic is composed of two categories, that is, “My name and my pronouns” and “One’s chosen gender.”

#### My Name and My Pronouns

This category refers to the name and pronoun that the participants felt truly identifies them. For them, it was crucial that the documentation to be completed in the Spanish public health system had the option of indicating the “chosen name” and that the healthcare employees addressed them in that way. They understood that the name that appears on their identity document is a legal identification, but that requirement should not rule out asking for the felt name as a non-exclusive option. Similarly, they claimed the need for health professionals to inquire about the pronoun with which they feel identified as this makes them feel visible.

Before the law, I call myself Ismael; it is like that, and I accept it. But I don’t feel like Ismael, nor do I identify with that name. When you go to the doctor, they should be concerned about making us feel good, and if they need to ask my legal name, let them do it. But let them also do it for the name and pronoun that I feel are really mine; both have a place. It is not very difficult to ask, “How do you want me to address you?” I believe that this simple question would open many doors (Heaven, 19).

I have never seen a box for the name felt when filling out any paperwork, neither in private nor in public. It is neither contemplated nor asked, and it does not matter. The other day, at the counter, I asked them to treat me with my name, which is not the legal one but the one I feel is really mine. The person who attended me was so surprised… Painful! (Fighter, 23).

#### One’s Chosen Gender

Similar to gender identification, this category refers to the gender that interviewees feel actually identifies them. For the interviewees, there were different genders away from the classic dichotomy of “masculine” and “feminine” among which they experience the connection and representation of the “non-binary gender.” They confirmed that no healthcare document had ever considered the option of marking “non-binary,” “other,” “alternative,” or any concept far from the traditional binarism. This situation bothered them, frustrated them, and they did not understand it; they perceived it as a situation of implicit institutional discomfort since they felt that the medical institution could not force them to position themselves between the only two possible options. This experience caused immense anguish to them.

It’s hard to claim that [having more than two gender options to check on medical documents] continuously because it’s conflicting. But at least you expect it from those people who take care of your health. I always claim it and do not check the box, but I understand that there will be many people who do not do it, who do not dare to do so. Whenever you go to a different place, you need to draw attention to the matter, it, and it’s an effort. It would be nice if everyone kept in mind that such a situation could occur, and I think that if it were facilitated… well, many more people would use it. They are not aware that this generates this violence, that coercion, that invisibilization… I mean conflicting because they make you feel different… The experience, well, is not pleasant (Ares, 42).

Well, I don’t know if it hurts, angers, or saddens me because health centers are supposed to be spaces that help you and look after you, and I can say that it is not like that. I am neither masculine nor feminine, neither she nor he. And they can’t force me to be what I’m not. I exist, so they should consider that they are doing something wrong when people like me are left out of that classification (Heaven, 19).

For the participants, the relationship that was apparently established between the anatomy, genitals, and assigned gender was a source of discomfort and misunderstanding since having a vulva or penis should not be synonymous with being or feeling female or male, respectively. The anticipation of being in an unpleasant, conflictive, or discriminatory situation was evident to the extent that the study participants chose not to visit health centers.

We are much more than a body. In the health centers, when they look at you, they will take care of you from the outside, that is, physically. They will see that I have a penis, and they will place me in the male category. And for me, that’s very aggressive because I must continually clarify my reality. No professional has ever asked me if this is so if I really feel that way. You don’t recognize what I really am; I don’t exist. My penis is worth more than me… And well, it’s not nice, and that’s why I don’t go to any health center. They have managed to isolate me (Rex, 23).

In the school textbooks, I never felt represented, but years have passed, and I continue to see a non-inclusive anatomy and a burden of genders that makes us very vulnerable and marginalized (Cielo, 19).

They anticipated negative reactions regarding the use of segregated spaces such as toilets in any health institution or the potential circumstance of getting admitted to a hospital. The interviewees claimed the option of neutral spaces in addition to spaces segregated by sex, indicated by a binary iconography. This duality would not only benefit non-binary people but also society as a whole because it would take into account the existing social plurality and would provide everyone with several options to choose from.

When you go to the toilet, it’s a joke. I look at the drawings of the sink and think that we live in the prehistoric era. This is very serious. It is as easy as the existence of a neutral space where those that already exist are not removed. It’s always about adding and not subtracting. The only neutral service that exists is for people with reduced mobility (Jerry, 29).

### I Don’t Exist for the Public Health Services

The second theme of the study refers to the fact that the participants perceived the general population and health providers, consider this population as non-existent. Participants emphasized that this situation of invisibility was the main cause of feeling excluded and vulnerable in a society that did not recognize them, which is consistent with the literature. This topic comprises two categories: “uneducated health system in sexual health” and “feeling like an outsider for being non-binary.”

#### Lack of Education in Sexual Health in the Health System

The interviewees perceived the lack of training of health providers, including misinformation and deficit of knowledge in relation to non-normative societal groups, as one of the main causes of their invisibility in the health context. They felt that there was a great deal of misinformation and that health providers lack the awareness and skills to deal with these situations. Not only they confirmed that healthcare workers did not know how to locate or treat them, but they felt that they had misconceptions about them, such as that they were homosexual or that they had not yet defined what gender attracts them. These professionals are merging concepts such as gender identity and sexual orientation. This misinformation meant that they must constantly embody an active pedagogy that included “teaching,” “explaining,” and “clarifying issues,” which made visits to the health center frustrating and exhausting. Participants perceived an urgent need for inclusion within the most training programs on gender, sexual health, and social diversity.

Change must happen from a young age at school when you are studying. It should occur in higher education. I studied pharmacy, and none of this was clarified. Well, seeing what has been seen, nothing is mentioned in other health careers because it is as if we did not exist. The person who attends me when I make an appointment at the primary health center does not even know what I am talking about when I ask him to address me by name. It’s not that he doesn’t want to, it’s just that he doesn’t know what I’m talking about. But honestly, I am tired of teaching those who should be trained in such matters, and it is exhausting (Gym, 39).

Additionally, participants reported that healthcare providers often held misconceptions about them, assuming they were homosexual, or unsure about their gender preferences. As one participant explained:

My nurse tells me that she understands me, but every time she calls me, she does so by the name that appears on my ID. I see that there is a great lack of training because it is not that she does not understand me; it is something that does not make sense to her. She doesn’t even know what I’m claiming. She thinks that I’m a lesbian and my problem is that I like women. That is why, it has been two years since I have set foot in the primary health center; it seems like a joke, but sadly, it is not (Lili, 22).

Overall, participants called for a more inclusive approach to training programs for healthcare professionals. They emphasized the need for education on gender diversity, sexual health, and social diversity. As one participant pointed out:

It’s very difficult to ask them to address you correctly, but it is even more challenging when they look at you as if they don’t know what you’re talking about. That leaves us with a bad impression as supposedly, they take care of our health. They do not need to be experts in the subject, but they do need to know that this can exist and not be taken by surprise (Black, 31).

#### Feeling Like an Outsider for Being Non-Binary

This category is defined as the awareness that the interviewees have of belonging to a minority social group compared to other social realities and prevailing societal norms. However, although despite the visibility of a much younger generation of non-binary adolescents (Kantor et al., 2023), the non-binary population is still uncommon, it does not imply that it should be hidden or discriminated against. All the participants confirmed that if there was more awareness of this phenomenon, they would more openly identify as non-binary because the existing misinformation means that many do not want to fight, show themselves as they are, or claim their place. If healthcare providers were more knowledgeable of and receptive to non-binary people’s identities, participants would be more likely to show up as their authentic selves and not avoid going to health centers. Participants in the study shared their experiences of growing up as non-binary individuals in small communities, where they keenly felt the isolating effect and the lack of recognition.

In my village, it was only me; it was like that. I had to leave because I felt like the weirdo, but when I went to primary health center, it was as if it did not exist, and it was much worse than at school. The fact that it was I was the only one in the village (or at least that’s what they said), that was no excuse for anyone to pretend like I didn’t exist. We are few, we are less, call it what you will, but we are and are in this world just like you. I am sure that if we were understood and respected more, I would have grown up with ‘more weird people like me (Dante, 34).

Additionally, a participant described their experience of having a “felt self” that exists outside the bounds of legal recognition. This ‘felt self’ remains hidden, contributing to a sense of being an outsider. Participants emphasized the potential benefits of accepting and accommodating non-binary individuals, calling for a collective shift in societal attitudes and perspectives.

Now I feel like there’s my real self and my legal self; imagine how I feel. It’s like my felt self doesn’t exist legally and is hidden. I know it would be very difficult to change, but we all must go in that direction. We would all benefit from the change, not just non-binary people but everyone. It takes formation and change to accept all that there is (Pam, 26).

## Discussion

Non-binary individuals face significant challenges in accessing inclusive healthcare services, highlighting a gap in the literature regarding their experiences and needs. This qualitative study demonstrates that non-binary people continuously attempt to be identified as they perceive themselves in all spaces, including health services. Their narratives highlight the necessity for a shift in diversity management and the creation of supportive social environments that affirm the rights and representation of diverse gender identities (Nicholas, 2019). Therefore, concrete actions, such as the use of their chosen name and pronoun, which often do not coincide with those that appear on their national identity document, are paramount.

Participants emphasize that the ability to choose their name and pronoun consistently fosters a trusting environment, aligning with Baldwin et al.’s (2018) findings on successful therapeutic relationships. However, the Catalan healthcare system inadequately supports the option to choose one’s preferred name and pronoun in health documentation and professional interactions, leading to substantial frustration and diminished confidence. The language used by non-binary individuals, as highlighted by Hansen and Żółtak (2022), may contribute to receiving negative perceptions, impacting the quality of healthcare. The lack of recognition of non-binary individuals’ names and needs induces discomfort and perpetuates negative perceptions, such as being perceived as challenging patients prone to mental health problems and disregarding their requests for medical transition, as noted by Lykens et al. (2018). This discomfort extends to contact with health centers, aligning with Taylor et al.’s (2019) emphasis on the invisibility and exclusion of non-binary gender in a dichotomous gender-taxing world.

This study reveals a crucial dimension in the way health providers link patients’ genitals, sex, and gender, potentially causing discomfort and misunderstandings. Reliance on cis-normative standards, along with the use of birth-assigned names and professionals’ confusion about sex-related concepts, may prompt individuals to avoid or delay healthcare visits, risking conflicts (Burchell et al., 2023; Jennings et al., 2019; Kcomt et al., 2020; Lerner et al., 2021; Lykens et al., 2018). Such delays can significantly impact health outcomes, particularly in cases like cancer where early diagnosis improves survival rates (Sociedad Española de Oncología Médica, 2022). Notably, certain pathologies are more prevalent among sexual minorities, heightening health risks for non-binary individuals due to structural avoidance of healthcare (Fredriksen-Goldsen et al., 2017; Scandurra et al., 2019). Health outcome disparities persist among cisgender, transgender, and gender-diverse individuals, with social stigma influencing these differences (Doyle et al., 2021; Feldman et al., 2016). This stress may contribute to higher rates of depression, suicidal tendencies, discomfort, and substance abuse among non-binary transgender individuals compared to their binary counterparts (Newcomb et al., 2020; Thorne et al., 2019). Despite their vulnerability, this study reveals non-binary individuals cease health center visits to evade misunderstandings and marginalization. Previous negative experiences contribute to a lack of trust in health providers, fostering reluctance to use services, enduring fear of discrimination, and perpetuating marginalization (Chung et al., 2021; Kcomt et al., 2020; Kearns et al., 2021; Teti et al., 2021).

Various studies substantiate challenges in accessing health centers for the LGBTIQ+ community. Quinn et al. (2015) highlighted how barriers and discrimination hinder healthcare access, impeding medical assistance-seeking. Santander-Morillas et al. (2022) revealed the negative emotional impact on trans individuals during healthcare interactions, leading to reluctance in exploring new facilities. Gonzales and Henning-Smith (2017) underscored discrimination and healthcare staff unawareness as primary barriers for non-binary individuals. Aparicio-García et al.’s (2018) pioneering Spanish study identified health indicators for non-binary individuals, revealing reduced support, limited community participation, and heightened mental health risks, highlighting multifaceted healthcare barriers. Age-related barriers are evident in Parra and Missé’s (2022) report on trans children, with 7–22% non-binary minors, and López-Sáez and Platero’s (2022) work during the COVID-19 pandemic, emphasizing less support and perceived burden among non-binary individuals compared to other LGBTIQ+ groups.

The experience for non-binary people is especially unpleasant when using gender-segregated spaces (such as toilets or shared rooms according to gender), or gendered clothing during hospital admissions, as Chandler (2020) confirms. Hence, one of the needs of the interviewees is the creation of inclusive spaces. Diminishing the discomfort non-binary people perceive from healthcare spaces is important, but also it can be an opportunity to rethink how hospitals are built on a binary division of spaces, and other sorts of grouping could be organized as an alternative (based on age, type of illness, severity, etc.) (Mikulak et al., 2021). This type of safe space could reduce the marginalization and segregation felt by people who do not identify with the classic gender dichotomy, not only for the non-binary population but also for the rest of LGBTIQ+ groups or other realities present in society (Kellett & Fitton, 2017; McNamara & Ng, 2016).

This study underscores the frequent exclusion, vulnerability, and lack of recognition faced by non-binary individuals, attributing these issues to their invisibility. It emphasizes the adverse effects of misinformation and inadequate training among healthcare providers concerning emerging social realities, corroborating Gonzales and Henning-Smith’s (2017) findings. Notably, sexuality education for healthcare professionals is insufficient and varies widely across university curricula (Ballering et al., 2023; Dalfó-Pibernat et al., 2015; Wickham et al., 2023), leading to discomfort and deficits in social skills when addressing patient issues and hindering the provision of holistic care (Doyle & Barreto, 2023; Klaeson et al., 2017; Leyva-Moral et al., 2021; Verrastro et al., 2020). This inadequacy is especially evident in training related to LGBTIQ+ groups (Nowaskie & Patel, 2020; Pichardo Galán & Puche Cabezas, 2019). Health sciences students recognize these deficiencies and advocate for more inclusive content addressing the specific needs of diverse groups (Liang et al., 2017). The integrative literature review by Norris and Borneskog (2022) reiterates the lack of knowledge among health professionals, highlighting the negative consequences for non-binary and trans individuals in their interactions with health centers. Consequently, a call for competence urges the abandonment of presumptions, stigmatizing attitudes, and the ongoing need for advocacy experienced by non-cis-normative groups within the healthcare environment.

Ensuring comprehensive formative content and mitigating negative experiences for non-binary individuals necessitate the adoption of inclusive language tailored to their needs, aligning with the person’s self-defined gender identity. Key strategies include refraining from assumptions about gender or sexual identities, soliciting information on preferred names and pronouns, and using inclusive anatomical terminology (Baldwin et al., 2018; Goldhammer et al., 2018; Laiti et al., 2019). The concept of “affirming care,” as defined by Paceley (2021), becomes pivotal in health practices tailored to diverse communities, particularly non-binary and transgender individuals. Affirmative spaces in the health system, encouraged by this care model, can alleviate existing barriers in gender-diverse care. Education emerges as a potent tool to address these challenges (Morris et al., 2019), with participants advocating for educational programs normalizing and understanding gender, sexuality, and social diversity.

McCann and Brown (2020) propose incorporating the needs of LGBTIQ+ individuals into nursing training to diminish health inequities and enhance their health experiences. However, studies reveal deficiencies in training hours devoted to LGBTIQ+ issues, with medical schools allocating an average of five hours (Sawning et al., 2017) and nursing schools reducing this to 2.12 h (Lim et al., 2015). Irwig (2016) indicates significant competence gaps among healthcare professionals, emphasizing the need for robust training in caring for trans individuals. Gasch-Gallén (2021) outlines advances and debates in training for sexual, bodily, and gender diversity in Health Sciences degrees, highlighting progress but acknowledging persistent gaps. The lack of knowledge and consideration toward diverse gender identities poses ethical concerns, potentially leading to adverse health outcomes and rights violations for non-binary individuals. Thus, an imperative shift in healthcare training curricula is essential to address these new realities, making inclusivity not an optional consideration but a mandatory element across all contexts. As mentioned previously, it is necessary to train professionals in sexual health issues using an approach that integrates sex and gender, rather than a solely biologicist approach. As health science professionals, we cannot neglect the biological aspects of the individuals we care for, but this cannot be the central element guiding the therapeutic relationship. A therapeutic balance must prioritize both the respect for gender identities and the allowance for gender expressions, while also considering the biological elements necessary for the practice of the health sciences (McGregor et al., 2019; Miller et al., 2013).

### Limitations

The main limitations of the study are that the type of sampling carried out means that only self-assured and motivated adult people participated, thus excluding non-binary people outside this spectrum, or underage. While personal contacts were initially used for study promotion and visibility, it is essential to note that only two final participants had direct connections with research team members. These two participants were not known to the interviewer or the data analyst. Participant selection was conducted independently and objectively using the snowball sampling technique to ensure diversity and avoid bias. In addition, the interviews were conducted in Spanish and translated into English by a professional bilingual translator. During the translation process, some details may have been lost. However, close monitoring of the translation and back-translation process by one of the group’s researchers, also bilingual, ensured that the process had minimal impact on the results. Finally, typical of qualitative studies, the findings of this study cannot be generalized, but they can be transferred to similar sociocultural contexts.

### Conclusions

Non-binary people encounter multiple barriers when accessing the health services that make them feel invisible, vulnerable, and marginalized, thus making it difficult for them to access health centers. This barriers entails the following consequences: more health problems, less adherence and follow-up, greater risk of suffering from mental health disorders, and less perceived social support.

Policy-level institutional changes in healthcare are imperative to address these barriers effectively. Moreover, healthcare systems can improve the experiences of non-binary populations if healthcare workers are educated on gender, sexuality, and new social realities. However, the training curricula of different Spanish universities in the field of health rarely include these topics. It must promote a more inclusive training, which would guarantee holistic and quality care to all people, irrespective of their way of identifying themselves. Healthcare providers must undergo training that goes beyond traditional education and includes destigmatization training to create a more welcoming and respectful environment for all people, regardless of their gender identity or sexual orientation. This comprehensive approach is essential to foster an inclusive healthcare system that can effectively address the unique healthcare needs of non-binary individuals. Similarly, it is essential to conduct more specific studies as well as include this population in other generalist studies to not make inferences from results that do not represent them.

## Data Availability

The authors confirm that the data supporting the findings of this study are available within the article and its supplementary materials.
